# Yak Pericardium as an Alternative Biomaterial for Transcatheter Heart Valves

**DOI:** 10.3389/fbioe.2021.766991

**Published:** 2021-11-08

**Authors:** Mingzhe Song, Zhenjie Tang, Yuhong Liu, Xinlong Xie, Xiaoke Qi, Qiying Wu, Zhenlin Jiang, Zhongshi Wu, Tao Qian

**Affiliations:** ^1^ Department of Cardiovascular Surgery, The Second Xiangya Hospital, Central South University, Changsha, China; ^2^ Engineering Laboratory of Hunan Province for Cardiovascular Biomaterials, Changsha, China

**Keywords:** transcatheter heart valves, yak pericardium, tissue thickness, immunogenicity, biomaterial

## Abstract

Transcatheter aortic valve implantation (TAVI) has received much attention and development in the past decade due to its lower risk of complication and infections compared to a traditional open thoracotomy. However, the current commercial transcatheter heart valve does not fully meet clinical needs; therefore, new biological materials must be found in order to meet these requirements. We have discovered a new type of biological material, the yak pericardium. This current research studied its extracellular matrix structure, composition, mechanical properties, and amino acid content. Folding experiment was carried out to analyze the structure and mechanics after folding. We also conducted a subcutaneous embedding experiment to analyze the inflammatory response and calcification after implantation. Australian bovine pericardium, local bovine pericardium, and porcine pericardium were used as controls. The overall structure of the yak pericardium is flat, the collagen runs regularly, it has superior mechanical properties, and the average thickness is significantly lower than that of the Australian bovine and the local bovine pericardium control groups. The yak pericardium has a higher content of elastic fibers, showing that it has a better compression resistance effect during the folding experiment as well as having less expression of transplantation-related antigens. We conducted *in vivo* experiments and found that the yak pericardium has less inflammation and a lower degree of calcification. In summary, the yak pericardium, which is thin and strong, has lower immunogenicity and outstanding anti-calcification effects may be an excellent candidate valve leaflet material for TAVI.

## Background

Transcatheter heart valve implantation is one of the greatest innovations in treating patients with valvular heart diseases in the 21st century ((Lüscher, 2019)). The estimated number of potential transcatheter aortic valve implantation (TAVI) candidates was approximately 18,000 in Europe and 90,000 in Northern America ((Durko et al., 2018)). It is estimated that there are 2,000,000 patients with aortic valve stenosis over the age of 75 in China, of which 400,000 patients have not undergone surgical thoracotomy and valve replacement surgery, meaning that the demand for TAVI surgery in China is relatively large ( (Coats and Bonhoeffer, 2007; Wang et al., 2019)). Initially indicated for elderly patients who are inoperable or are at an extreme risk for conventional surgical valve replacement. TAVI has recently demonstrated non-inferiority for patients at low surgical risk in a short-term follow-up, as well as younger patients with much longer life expectancy ((Leon et al., 2010; Mack et al., 2019)). In this context, the long-term functionality and durability of bioprosthetic valves for TAVI have gained significant importance ((Fukuhara et al., 2020)). Several notions that are specific to transcatheter valves raise concerns about their durability.

First, the transcatheter implantation technique sets a limitation on the thickness of the valve. In efforts to reduce the procedure-related vascular complications, the size of the delivery system is continuously being reduced, thus requiring thinner leaflet materials. The commercial transcatheter aortic valves are derived from porcine (Medtronic CoreValve™) or bovine pericardium (Edwards SAPIEN™; Boston Scientific Lutos™), and have thinner leaflets than the surgical bioprostheses valves (∼0.25 vs ∼0.4 mm) (Arsalan and Walther, 2016). Although the long-term performance of TAVI remains unknown, an *in vitro* fatigue simulation study proved that the thinner valve leaflet material has worse durability compared to the regular thickness ( (Martin and Sun, 2015)). The stress of the leaflets and the stress-related calcification after being implanted increase with decreases in thickness (Abbasi and Azadani, 2017; Kostyunin et al., 2020). Furthermore, thin leaflets are associated with poor biostability and rapid degeneration (Costa et al., 2019). Second, the valves need to be folded over a balloon (balloon-expandable) or be folded within a sheath (self-expanding) to allow for transcatheter delivery. Several studies have proven that folding is associated with collagen structural destruction (Alavi et al., 2014), mechanical property degradation (Khoffi and Heim, 2015), and leaflet calcification (Zareian et al., 2019).

Bovine and porcine pericardium are currently utilized to make old fashion transcatheter valves. The bovine pericardium has optimal mechanical properties, while porcine pericardium has a lower thickness (Gauvin et al., 2013). Previous studies have investigated the bovine age-dependent differences of the pericardium tissues (Sizeland et al., 20142014; Caballero et al., 2017). However, the comparison of pericardia from different cattle breeds has not been reported. The domesticated yak (Bos grunniens, referred to as yak) is a native cattle of the Qinghai-Tibetan Plateau and is known as the roof ridge of the world, having harsh climates, hypoxia, and low atmospheric pressure. Compared with other cattle breeds, yaks usually weigh less but have an excellent working endurance and cardiopulmonary function.

In our previous research, we found that the yak pericardium has a thinner and denser overall structure. We hypothesized that the yak pericardium might serve as a novel biomaterial for use as a transcatheter heart valve. Herein, we compared the structural and mechanical properties, as well as the folding resistance of yak pericardium with other pericardia from two commonly employed cattle breeds and with porcine pericardium. At present, the biological heart valve materials in clinical use are all glutaraldehyde crosslinked products. We also explored the pericardial materials crosslinked by glutaraldehyde in order to find more suitable materials for making transcatheter heart valves.

## Materials and Methods

### Materials

Fresh yak pericardium (YP) was provided by NewMed Medical Co., Ltd. (Shanghai, China). Fresh Australian bovine pericardium (AP) (Hybridization between Bos Taurus and Bos indicus ) was provided by Cingular Biotechnology Co., Ltd. (Shanghai, China). Fresh local bovine pericardium (LP) was harvested from Chinese yellow cattle (Bos taurus) at a local slaughterhouse (Changsha, China). Fresh porcine pericardium (PP) was provided by Med-Zenith Medical Scientific Co., Ltd. (Beijing, China). 20 independent samples (derived from individual animals) were tested for each tissue source.

The fresh pericardia were immersed in cold 0.9% saline solution and kept on ice during transportation to the laboratory.

The selected pericardium patches were preserved in PBS (phosphate buffered saline). We used a magnifying glass to observe whether there were any apparent cuts, to cut off all areas that do not meet the necessary requirements, to carefully remove the fatty tissue on the material matrix with scissors and tweezers, and to grasp the material in parallel with the tweezers in order to avoid damaging the pericardium. After preparation, the pericardia were stored in ice for further use.

### Thickness and Water Content

Small patches of the native samples (1 cm × 1 cm, for each pericardium) were washed with deionized water and blotted dry. The thickness of each sample was measured at 3 different positions via a thickness gauge and the average value was recorded (*n* = 12). Water content was measured by weighting the sediment before and after desiccation by freeze-drying (*n* = 6) (−57°C for 24 h).

### Quantitative Biochemistry

Small patches of fresh pericardia samples (1 cm × 1 cm, *n* = 6 for each pericardium) were used for all biochemical analyses.

Collagen content was determined using a total collagen kit (Nanjing Jiancheng Co. Ltd. China) based on the detection of hydroxyproline. Elastin content was quantified using a Fastin assay (Biocolor Ltd., United Kingdom). Glycosaminoglycans (GAG) content was measured using a dimethyl-methylene blue colorimetric quantitative kit (Genmed Scientifics Inc, United States). All assays were conducted via the manufacturer’s protocol.

### Glutaraldehyde Crosslinking

Fresh pericardia were crosslinked using 0.625% glutaraldehyde (GA) in a 0.05 mM polybutylene succinate (PBS, PH 7.4) buffer at room temperature with gentle shaking for 24 h. Subsequently, crosslinking was continued with 0.2% GA in 0.05 mM PBS (PH 7.4) at room temperature for at least 6 days.

### Determination of the Degree of Cross-Linking

The free amino acid groups were determined using the ninhydrin experiment. The glutaraldehyde cross-linked pericardium of each group was cut into 0.5*0.5 cm sections, freeze-dried for 24 h, and reacted with 1% ninhydrin ethanol solution in a boiling water bath for 20min. After cooling, the supernatant was measured with a microplate reader at 570 nm and the OD values were recorded. Glycine was used as a standard control to calculate the degree of cross-linking.

### Light Microscopy

The fresh pericardia and GA-crosslinked pericardia were stored in formaldehyde, processed, embedded in paraffin, sectioned at 5 mm, and analyzed using light microscopy. Hematoxylin and eosin (HE), elastic van-Gieson (EVG), Alcian Blue, and Masson trichrome staining were used to visualize and assess the cell nuclei (blue-black), elastin network (black), Glycosaminoglycans (sky blue), and collagen fibers (blue), respectively.

### Scanning Electron Microscopy

Sample preparation was performed by fixing the samples using a 3% glutaraldehyde (2 h, 4 °C) solution. Washed three times with phosphate buffer solution/10min at 4 °C, then dehydrated using a 30, 50, 70, 90, 100, 100, and 100% alcohol for 10 min each. The samples were then soaked in tert-butanol for 2 hours. Next, the samples were placed into a freeze dryer (Thermo, ELECTRON CORPORATION, 450 Fortune Blvd. Milford MA 01757) for 24 h to dry. Finally, the samples are sprayed with gold and placed under a scanning electron microscope (Quanta 250 FEG, FEI company, Czech Republic) for sample observation.

### Protein Extraction

The fresh pericardia and GA-crosslinked pericardia underwent manual mincing and incubation in a protein extraction solution containing 0.1% sodium dodecyl sulfate SDS) at 1,000 rpm, 4°C for 1 h. Following centrifugation, the supernatant was recovered. All extracts were stored at −80°C.

### One-Dimensional Electrophoresis and Western Blot

Protein extracts were assessed for antigenicity using a one-dimensional electrophoresis and Western blot. Selected murine anti-Gala1-3Gal b1-(3)4GlcNAc-R (alpha-gal) (Enzo Life Sciences, Farmingdale, NY) and Anti-Neu5Gc Antibody Kit (BioLegend Way, San Diego, CA) to detect fresh pericardium protein. Loading controls for all western blot images are protein extraction solution volume, with equal volumes loaded in each well, equating to loading of equal starting tissue mass per well (*n* = 3 per group) (Dalgliesh et al., 2018). Densitometry was determined using Image J acquisition and analysis software, with all lanes corrected for background. The antigen expression levels are based on fresh LP used as the baseline, and the ratio of the remaining samples.

### Enzymatic Degradation Testing

The collagen and elastin stabilities of the fresh pericardia were studied using a collagenase and elastase treatment. Six samples of each group were placed into individual microfuge tubes, lyophilized (Thermo,ELECTRON CORPORATION,450 Fortune Blvd.Milford MA 01757), and weighed. Then the samples were incubated in Tris buffer (Tris 0.1 M, CaCl2 0.05 M, pH = 7.4) containing 125 U/ml collagenase IA (C2674-1G, Sigma) or 30U/ml elastase (E1250-50MG,Sigma) for 24 h at 37 °C while shaking at 100 RPM. The resulting washed samples were lyophilized and re-weighed. Data were calculated as a percent loss from the original dry sample’s weight.

### Cell Culture and Cytotoxicity Assessment

Human umbilical vein endothelial cell line (EAhy926) were culture in high glucose Dulbecco’s Modified Eagle Medium with 10% fetal bovine serum (DMEM/10%FBS) . The sample were of 1 × 1 cm^2^ cut from fresh samples and GA-crosslink samples and cultured in DMEM/10%FBS at 37°C for 24 h at a density of 2.5 ml/cm^2^ . The culture media (leach liquor) were collected and preserved. The media were replaced with leach liquor from the sample cultures diluted with DMEM/10%FBS at ratios of 1:2. The cells were cultured for a further 1, 3 and 5 days at 37°C. A negative control was prepared using DMEM/10%FBS alone. The mitochondrial metabolic (MTT) assay was applied in assessing the cell growth on the sample. The optical density at 570 nm was determined using a microplate reader. The cytotoxicity of each protocol was evaluated by calculating the relative growth rate (RGR), RGR = (mean OD for each group)/(mean OD of the negative control) × 100% to determine the proliferation index (Xu et al., 2017).

### Amino Acid Content Detection

Using 100 mg of each sample (*n* = 3) an aliquot of each sample was precisely weighed and transferred to an Eppendorf tube. After addition 1,000 *μ*l of extraction solution (precooled at −20°C, acetonitrile-methanol-water, 2:2:1, containing the isotopically-labeled internal standard mixture), the samples were vortexed for 30 s, homogenized at 40 Hz for 4 min, and sonicated for 5 min in an ice-water bath. The homogenization and sonication process was repeated 3 times, followed by incubation at −40°C for 1 hour and centrifugation at 12,000 rpm (RCF = 13,800 (×g), R = 8.6 cm) for 15 min at 4°C. An 80 *μ*l aliquot of the clear supernatant was transferred to an auto-sampler vial for UHPLC-MS/MS analysis.

The UHPLC separation was carried out using an Agilent 1,290 Infinity II series UHPLC System (Agilent Technologies), equipped with a Waters ACQUITY UPLC BEH Amide column (100 × 2.1 mm, 1.7 *μ*m). An Agilent 6460 triple quadrupole mass spectrometer (Agilent Technologies), equipped with an AJS electrospray ionization (AJS-ESI) interface, was applied for assay development. An Agilent MassHunter Work Station Software (B.08.00, Agilent Technologies) was employed for MRM data acquisition and processing.

### Thermal Stability Testing

For thermal stability testing, we cut each sample group into 1 cm*5 cm strips (*n* = 8). Using distilled water as the medium, we heated the samples starting at 20°C and increased the temperature by 5°C per minute. Finally, we used an HG-1 leather shrinkage temperature tester (Sichuan Chengdu Dachengxing Digital System Co., Ltd.) to measure the shrinkage temperature.

### Mechanical Testing

For mechanical testing, we cut each group of materials into 1 cm*5 cm strips. Using an electronic tensile testing machine (Instron, United States, electronic universal material testing machine), we measured and recorded each sample’s thickness and stretch length. Setting the tensile rate to 100 mm/min, we calculated the material’s thickness (mm), elastic modulus (MPa), ultimate tensile stress (MPa), maximum load (N), and strain failure (%).

### Folding Experiment

The Folding experiment was tested in each sample group using 5 cm*5 cm (*n* = 6) sections. Next, we folded the pericardium symmetrically twice, and applied a specific amount of pressure on each folded pericardium (100N,1 h) to simulate the environment of catheter placement.

### Subcutaneous Implantation in Rats

The animal experiments were authorized by the Second Xiangya Hospital of Central South University Animal Experiment Ethics Committee and Authority for Animal Protection. All animal experiments complied with the ARRIVE guidelines and were carried out in accordance with the United Kingdom. Animals (Scientific Procedures) Act, 1986, and associated guidelines. The model in this study was a subdermal implantation using SD rats (4–5 Weeks, Male, 80–120 g, *n* = 5). After the anesthesia process, four different pericardia (10*10 mm, *n* = 5) samples were inserted into two 1.0 cm dorsal incisions of each rat. The incisions were finally sutured using polypropylene 3-0. All rats were sacrificed using the de-neck method on the 56th day. The specimens were then removed and stored at −80°C for subsequent experiments.

### Calcium Content Detection

For the analysis of calcium content, the pericardia samples were removed from the rats. The samples were then digested using 6 M HCl. A calcium content detection kit (Abcam) was used to quantify the pericardia’s calcium content, and detected with an OD value of 560 nm.

### Histological and Immunological Analysis

The histological and immunological analyses of the tissue samples were conducted after the dehydration of the samples. The samples were then embedded in paraffin and sectioned to a thickness of 3 µm for staining. The H&E staining shows the cells and tissue morphology. VONKASSA staining verifies the calcium deposits within the implanted samples.

For immunohistochemistry (IHC) and immunofluorescence (IF) staining, the sections were deffinity and rehydrated before antigen extraction. The sections were incubated with the primary antibody overnight at 4°C. A rabbit anti-rat CD68 antibody (Servicebio Co. Ltd. Wu Han China; dilution 1:500) was used to label M1 macrophages (brown) and a rabbit anti-rat CD206 antibody (Abcam; dilution 1:1,000) was used to label M2 macrophages (red).

### Statistical Analysis

Data were reported as the means ± standard deviations (*n* ≥ 3). One-way and two-way analysis of variance (ANOVA) and standard Student’s t test was used to determine the significance of the difference between the group, and a value of *p* < 0.05 was considered statistically significant.

## Results

### Morphology and Chemical Composition

**FIGURE 1:**
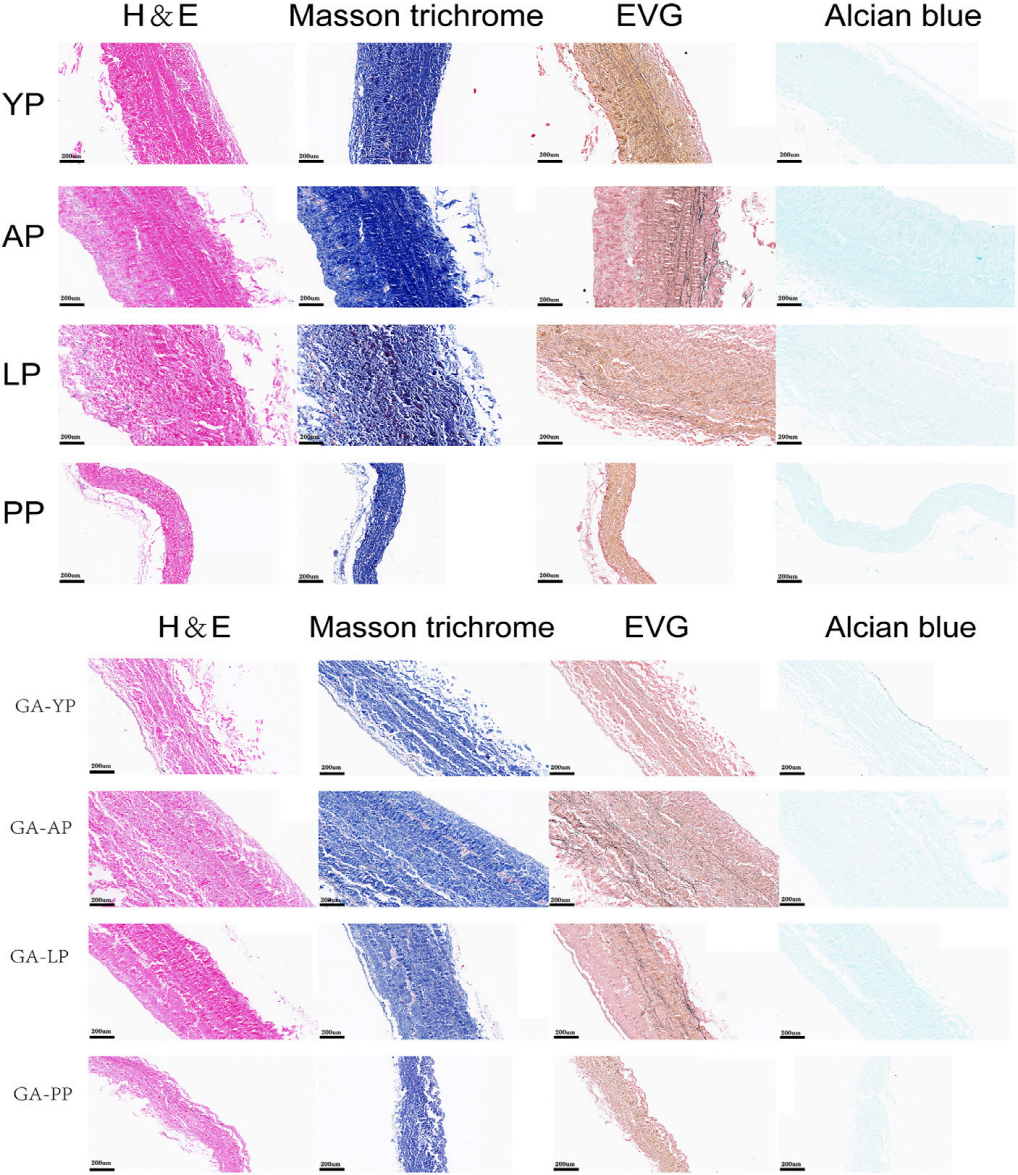
HE staining, MASSON staining, EVG staining, and Alcian blue staining for ECM evaluation of fresh yak pericardium, fresh Australian bovine pericardium, fresh local bovine pericardium, fresh porcine pericardium ,GA-crosslinked yak pericardium, GA-crosslinked Australian bovine pericardium, GA-crosslinked local bovine pericardium, and GA-crosslinked porcine pericardium.scale bars are 200 um.

The histological staining of the fresh and GA-fixed pericardia is shown in Figure 1. The pericardia consisted of collagen, elastin, and GAG. The yak pericardia (YP) have regularly arranged wavy collagen fibers, and evenly distributed straight elastic fibers. Both the collagen and elastic fibers remained intact after GA crosslinking.

### Chemical Composition.

**FIGURE 2:**
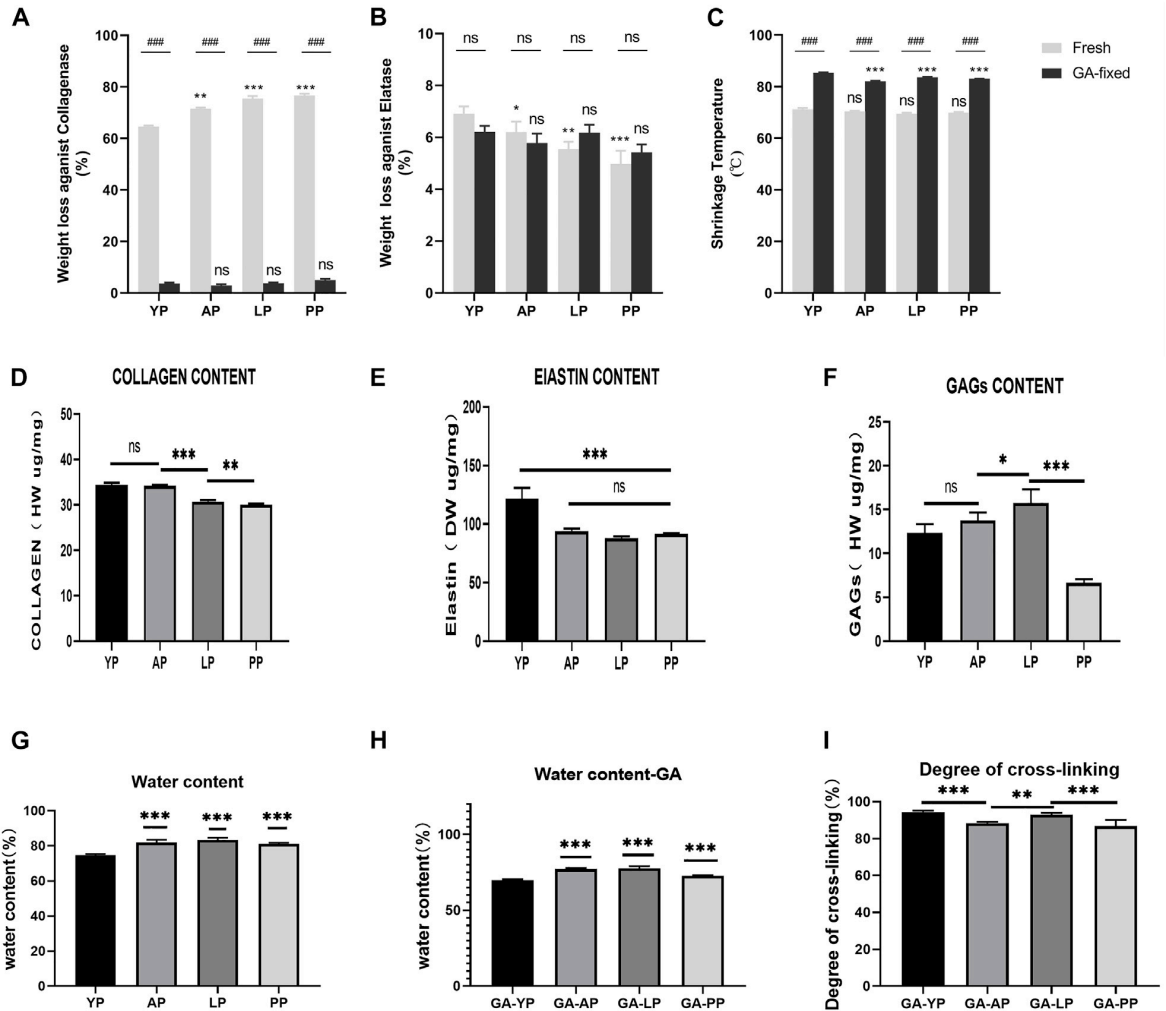
Enzymatic degradation and Thermal stability. **(A)**, Fresh and GA-crosslinked pericardium weight loss against collagenase; **(B)**, Fresh and GA-crosslinked pericardium weight loss against Elatase; **(C)**, Fresh and GA-crosslinked of shrinkage temperature. Enzymatic degradation :(*n* = 6,*p < 0.05; **p < 0.01;***p < 0.001; ns represents no significant difference). Thermal stability: (*n* = 8,*p < 0.05; **p < 0.01;***p < 0.001; ns represents no significant difference) Evaluation of water content,degree of cross-linking, collagen, elastin, and GAGs of fresh and glutaraldehyde cross-linked pericardium. **(D)**, collagen content of fresh pericardium; **(E)**, elastin content of fresh pericardium; **(F)**, GAGs content of fresh pericardium. **(G)**, water content of fresh pericardium; **(H)**, water content of GA-crosslinked pericardium; **(I)**, degree of GA-crosslinked pericardium. (*n* = 6; *p < 0.05; **p < 0.01;***p < 0.001; ns represents no significant difference)

Compared with other pericardia, YP has the lowest water content before and after GA crosslinking (*p* < 0.001, Table 1, Figure 2). As shown in Figure 2, the collagen content of fresh YP was similar to AP (*p* > 0.05), but significantly higher than LP (*p* < 0.001) and PP (*p* < 0.001). The YP has the highest effective elastin content compared to other pericardia (*p* < 0.001). The GAG content of the YP was similar to AP (*p* > 0.05), and was significantly lower than LP (*p* < 0.001); however, it was higher than PP (*p* < 0.001).

**TABLE 1:** Morphology characteristics and mechanical properties for the 4 pericardia tissues.

	YP	AP	LP	PP
Thickness (mm)				
Fresh (*n* = 8)	0.34 ± 0.05	0.53 ± 0.07	0.53 ± 0.06	0.26 ± 0.05
GA-fixed (*n* = 8)	0.38 ± 0.04	0.58 ± 0.05	0.57 ± 0.05	0.27 ± 0.02
Water content (%)				
Fresh (*n* = 8)	74.6 ± 0.2	81.9 ± 0.5	83.3 ± 0.5	81.0 ± 0.4
GA-fixed (*n* = 8)	69.9 ± 0.3	77.2 ± 0.3	77.6 ± 0.6	72.8 ± 0.2
Collagen content (ug/mg)HW	34.4 ± 0.5	34.2 ± 0.2	30.7 ± 0.3	30.1 ± 0.2
Elastin content (ug/mg)DW	121 ± 9.2	94.8 ± 2.06	88.26 ± 1.26	91.93 ± 0.28
GAG content (ug/mg) HW	12.3 ± 1.0	13.8 ± 0.9	15.8 ± 1.5	6.6 ± 0.4
Weight loss after collagenase digestion (%)				
Fresh (*n* = 6)	64.6 ± 1.0	64.5 ± 1.2	75.4 ± 2.6	76.7 ± 1.5
GA-fixed (*n* = 6)	3.6 ± 0.9	2.9 ± 1.1	3.8 ± 0.8	5.0 ± 1.2
Weight loss after elastase digestion (%)				
Fresh (*n* = 6)	5.9 ± 0.7	8.2 ± 1.0	5.6 ± 0.7	3.6 ± 1.2
GA-fixed (*n* = 6)	6.2 ± 0.6	5.8 ± 0.9	6.2 ± 0.8	7.2 ± 0.8
Elastic modulus (MPa)				
Fresh	153.8 ± 34.5	121.6 ± 29.5	168.2 ± 31.8	126.1 ± 22.2
GA-fixed	156.5 ± 32.7	134.1 ± 30.2	163.6 ± 34.0	132.3 ± 30.3
Failure strain (%)				
Fresh	15.6 ± 3.7	16.7 ± 3.3	17.1 ± 2.7	15.0 ± 3.6
GA-fixed	28.4 ± 4.5	23.5 ± 4.5	20.2 ± 3.2	15.7 ± 3.6
UTS (MPa)				
Fresh	15.8 ± 5.8	11.8 ± 4.1	13.1 ± 4.2	10.8 ± 2.2
GA-fixed	15.9 ± 5.3	17.2 ± 3.9	19.0 ± 5.0	12.2 ± 3.9
Maximum load (N)				
Fresh	53.0 ± 21.3	68.0 ± 31.0	77.6 ± 6.7	28.2 ± 8.5
GA-fixed	54.5 ± 19.7	104.3 ± 22.8	106.0 ± 23.8	32.6 ± 11.0
Ca content (ug/mg)	2.63 ± 1.31	50.9 ± 13.95	104.5 ± 10.7	26.85 ± 10.5
Fibrous sac (um)	22.53 ± 14.18	44.32 ± 31.78	39.68 ± 21.58	44.66 ± 28.55
	Fold-YP	Fold-AP	Fold-LP	Fold-PP
Thickness (mm)	0.39 ± 0.04	0.69 ± 0.13	0.64 ± 0.12	0.21 ± 0.02
Elastic modulus (MPa)	164 ± 42.24	133.77 ± 19.7	136.9 ± 24.78	113.2 ± 33.3
Failure strain (%)	19.29 ± 3.28	23.5 ± 2.64	16.09 ± 6.5	10.96 ± 2.7
UTS (MPa)	20.46 ± 5.48	16.83 ± 3.63	14.36 ± 6.38	11.55 ± 2.67
Maximum load (N)	62.54 ± 23.22	80.45 ± 34.9	80.47 ± 58.06	15.59 ± 6.59

### Amino Acid Content Detection

**FIGURE 3:**
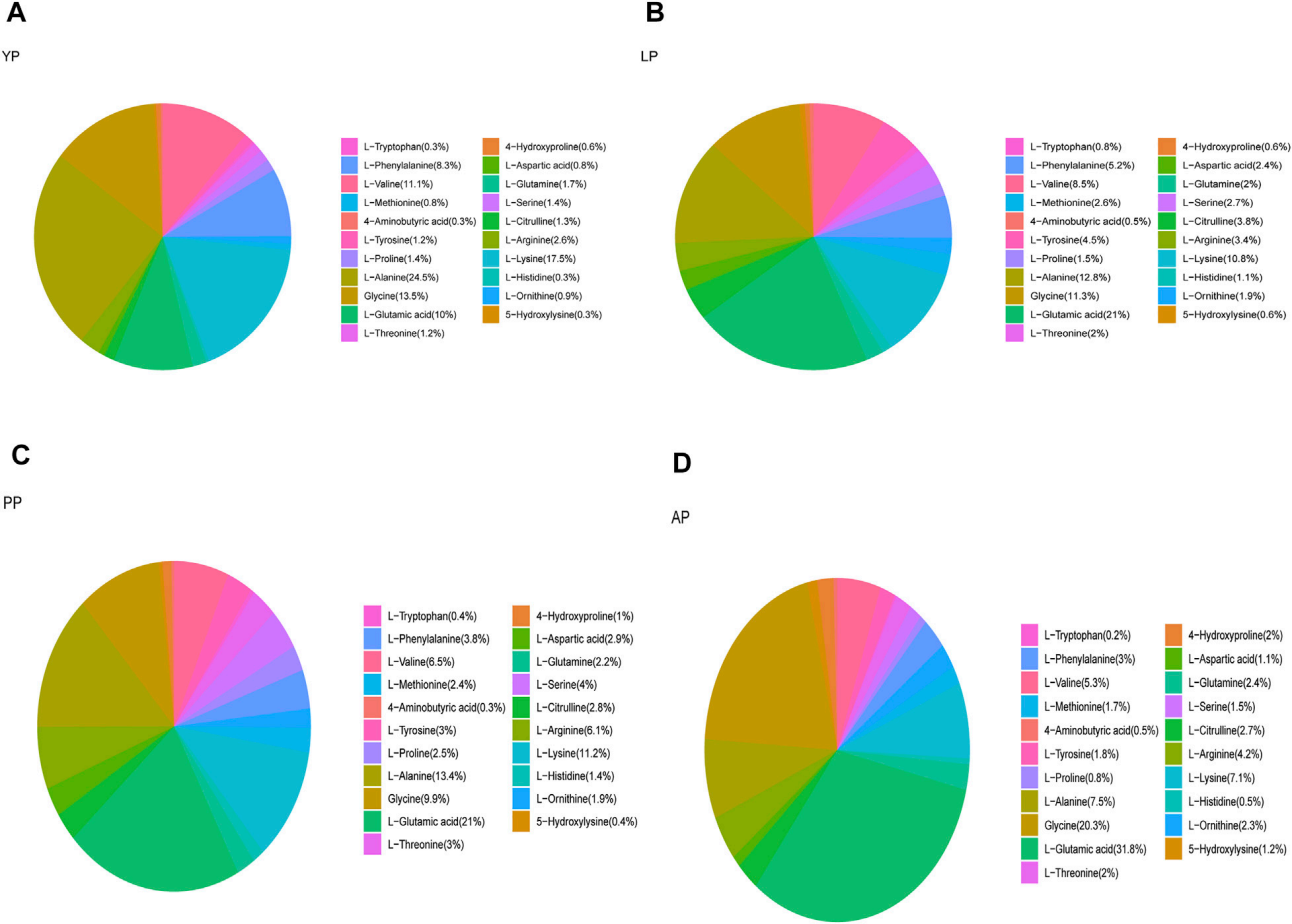
We detected the amino acid content of all fresh pericardium and count the proportion of amino acids in each group of pericardium. **(A)**, Fresh yak pericardium (YP) amino acids; **(B)**, Fresh local bovine pericardium (LP) amino acids; **(C)**, Fresh porcine pericardium (PP) amino acids; **(D)**, Fresh Australian bovine pericardium (AP) amino acids.

**FIGURE 4:**
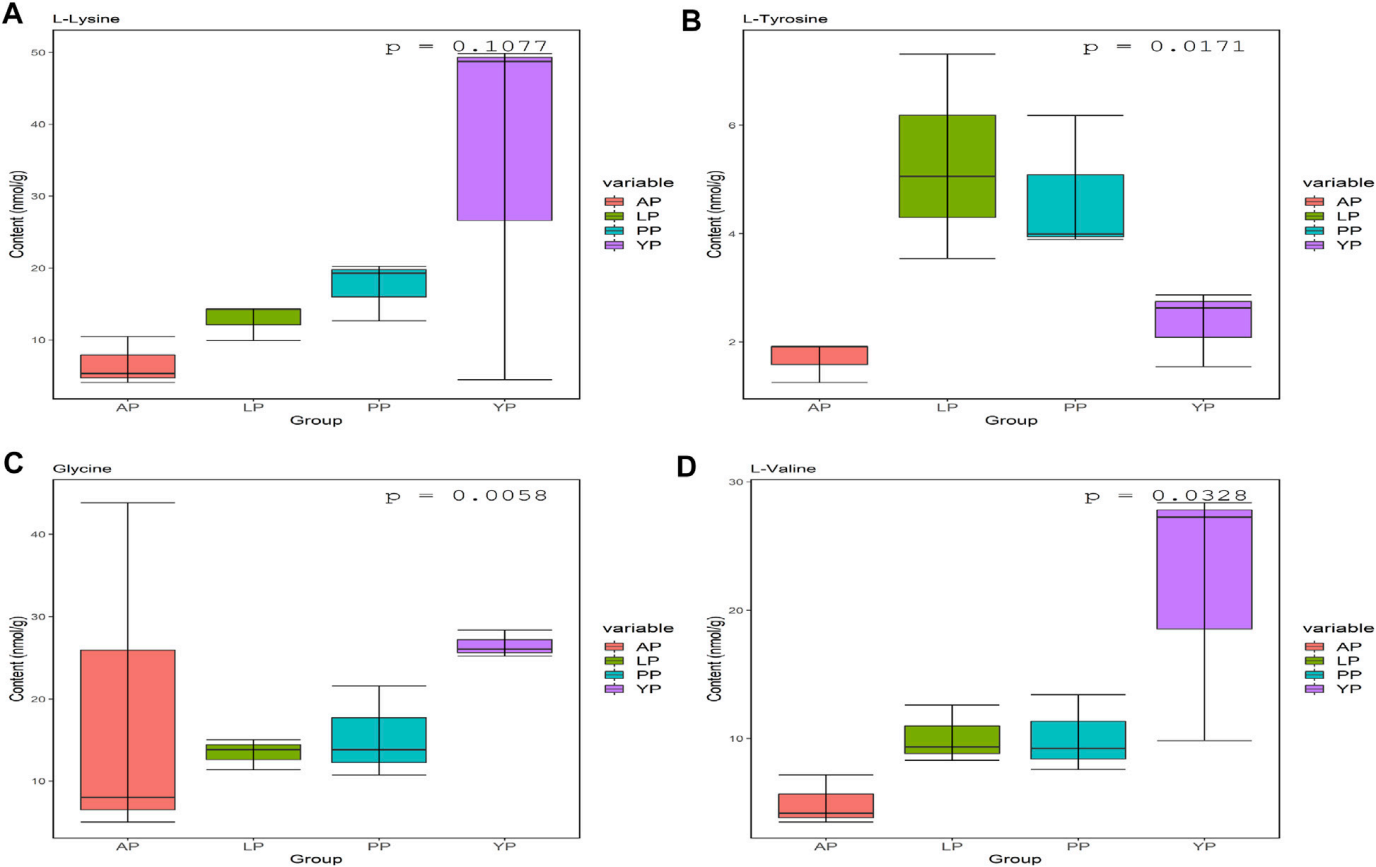
Lys **(A)**, Tyr **(B)**, Gly **(C)** and VAL **(D)** contents (nmol/g) in fresh yak pericardium (YP); fresh Australian bovine pericardium (AP); fresh local bovine pericardium (LP) and fresh porcine pericardium (PP).

We analyzed the amino acid content of the four pericardia groups and found that the amino acid composition of the different pericardium is slightly different. Alanine is the most abundant in the yak pericardium, and glutamate is the most abundant in the other three groups of pericardium (Figure 3, Supplementary Table S1). We analyzed several amino acids related to the production of the biological valves (Figure 4). The cross-linking of glutaraldehyde primarily relies on the amino side chain group of lysine for cross-linking (Jorge-Herrero et al., 2005). The lysine content of the yak pericardium is greater than the other three groups of pericardia, suggesting that the yak pericardium is more suitable for cross-linking with glutaraldehyde, This was verified in our previous GA cross-linking test. We also analyzed glycine and valine. The glycine and valine in the yak pericardium are significantly higher than in the other three groups. These two amino acids are related to the hydrophobic structure of elastin, and the results are consistent with the detection of the elastin content (Han et al., 2003). Finally, we analyzed tyrosine. The triple helix structure of collagen is evolutionarily conserved; therefore, the collagen of different organisms is very similar. However, the terminal peptide sequences of collagen in humans and other animals are significantly different. Collagen containing biomaterials implanted into the human body easily produce an immune response caused by the terminal peptide. Moreover, tyrosine only exists in the terminal peptide of collagen; thus, a significant decrease in tyrosine means the blocking of the terminal peptide and a decrease in immunogenicity (Geiger and Friess, 2002). The tyrosine content of the YP is significantly lower than in the other three pericardia. This result suggests that the immunogenicity of the yak pericardium may be lower than the other pericardium.

### Enzymatic and Thermodynamics Stability

Enzymatic stability. As presented in Table 1 and shown in Figures 2A,B, fresh pericardial tissues were all degraded in 24 h losing over 60% of their dry weight after an incubation in collagenase and losing less than 10% after incubation in elastase after 24 h. However, the fresh YP lost significantly less weight than AP (*p* < 0.01), LP (*p* < 0.001), and PP (*p* < 0.001) using collagenase, however lost significantly more weight than AP (*p* < 0.05), LP (*p* < 0.01), and PP (*p* < 0.001) using elastase. GA crosslinking drastically decreased the collagenase degradation of the pericardia (*p* < 0.001) having a weight loss of less than 5.0%; however, it had no significant effect on the elastase degradation (*p* > 0.05). Furthermore, after crosslinking with GA, no significant difference in weight loss was found among YP, AP, LP, and PP using either collagenase or elastase.

### Thermodynamics Stability.

As shown in Figure 2C, the denaturation temperature (DT) of fresh YP was 71.2°C, similar to AP, LP, and PP. GA crosslinking significantly increased the DT of the pericardia (*p* < 0.001). The DT of GA-fixed YP was 85.3°C, which was significantly higher than the GA-fixed AP, LP, and PP (*p* < 0.001).

### Degree of Cross-Linking

We tested the degree of GA-crosslinking in each pericardium (Figure 2), YP has a higher degree of crosslinking with glutaraldehyde, which corresponds to our amino acid test.

### Biocompatibility *in vitro*

The biocompatibility of groups of samples before and after GA cross-linking was determined by MTT assay.As shown in (Supplementary Figure S1), the relative growth ratios (RGRs)of HUVECs were grown in the presence of leach liquor from the sample at concentrations after 1, 3, and 5 days of culture were evaluated. On day 1, no significant difference was observed in the RGR between the groups; on day 3 and day 5, the RGRs of each group reached level 1 (RGRs<75%), and there was no significant difference between the GA cross-linked and fresh groups.

**FIGURE 5:**
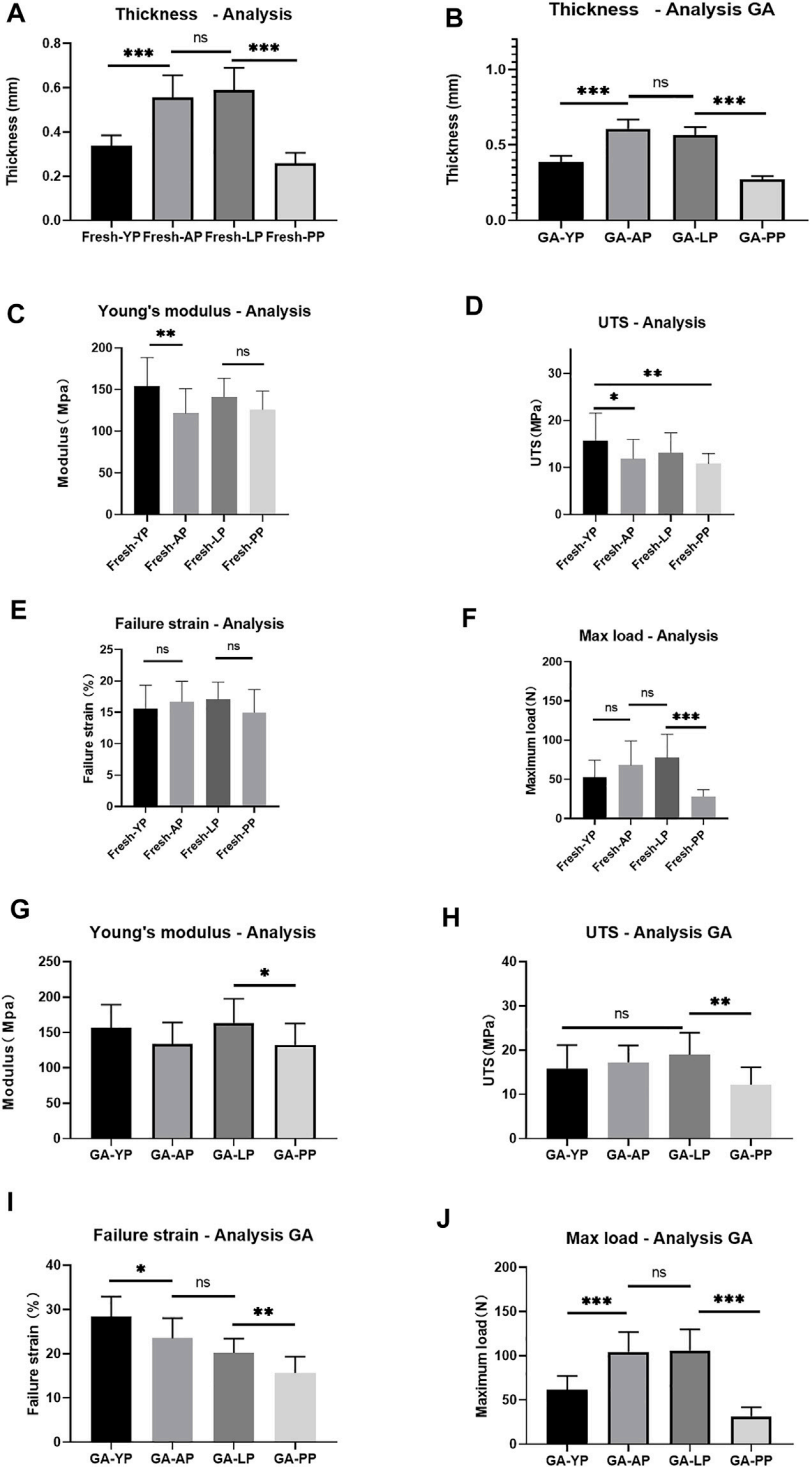
**(A)**, Detect the thickness of fresh pericardium. **(B)**, Detect the thickness of GA-crosslinked pericardium. (*n* = 12; *p < 0.05; **p < 0.01;***p < 0.001; ns represents no significant difference). Mechanical performance of fresh yak pericardium (Fresh-YP), fresh Australian bovine pericardium (Fresh-AP), fresh local bovine pericardium (Fresh-LP), and fresh porcine pericardium (Fresh-PP). **(C)**, Young’s modulus; **(D)**, The ultimate tensile strength (UTS). **(E)**, Failure strain; **(F)**, Max load. (*n* = 20; *p < 0.05; **p < 0.01;***p < 0.001; ns represents no significant difference) Mechanical performance of GA-crosslinked yak pericardium (GA-YP), GA-crosslinked Australian bovine pericardium (GA-AP), GA-crosslinked local bovine pericardium (GA-LP), and GA-crosslinked porcine pericardium (GA-PP). **(G)**, Young’s modulus; **(H)**, The ultimate tensile strength (UTS). **(I)**, Failure strain; **(J)**, Max load. (*n* = 10; *p < 0.05; **p < 0.01;***p < 0.001; ns represents no significant difference).

Thickness. As presented in Table 1, the mean thickness of fresh YP was 0.34 ± 0.01 mm, which was significantly thinner than AP and LP, and thicker than PP (*p* < 0.001, Figure 5). After crosslinking with GA, the thickness of the YP slightly increased to 0.38± 0.04 mm. The thickness increase did not reach statistical significance after crosslinking all of the pericardia (Ps > 0.05).

### Mechanical Properties

We performed mechanical testing on the fresh pericardium and the results are shown in Table 1 and Figure 5. YP had a higher Young’s modulus than AP (*p* < 0.01) and PP (153.8 ± 34.5 MPa vs 121.6 ± 29.5 MPa vs 126.1 ± 22.2 MPa) (*p* < 0.05) (Figure 5C). All pericardia have similar strain failures (Figure 5E); however, after cross-linking with GA, the strain failure of the yak pericardium was significantly improved compared with the other pericardia’s (28.4 ± 4.5%) (Figure 5I) (*p* < 0.001). Fresh YP (15.8 ± 5.8 MPa) has similar UTS with LP (13.1 ± 4.2Mpa) but has a higher UTS than AP (11.8 ± 4.1Mpa) and PP (10.8 ± 2.2Mpa) (Figure 5D) (*p* < 0.05;<0.01). Furthermore, after glutaraldehyde cross-linking, LP (19.0 ± 5.0 Mpa) was significantly improved and was higher than PP(12.2 ± 3.9 Mpa) (*p* < 0.001); however, no significant difference was observed in the AP (17.2 ± 3.9 Mpa) and YP (15.9 ± 5.3Mpa) groups (Figure 5H) (*p* > 0.05; >0.05). Whether it was the cross-linked glutaraldehyde or the fresh YP, the max load was significantly lower than the AP and LP groups (Figures 5F,J); this is due to its thickness being significantly thinner than the two groups. If the thickness is the same, the max load of the YP may be better than the others.

### Scanning Electron Microscopy

**FIGURE 6:**
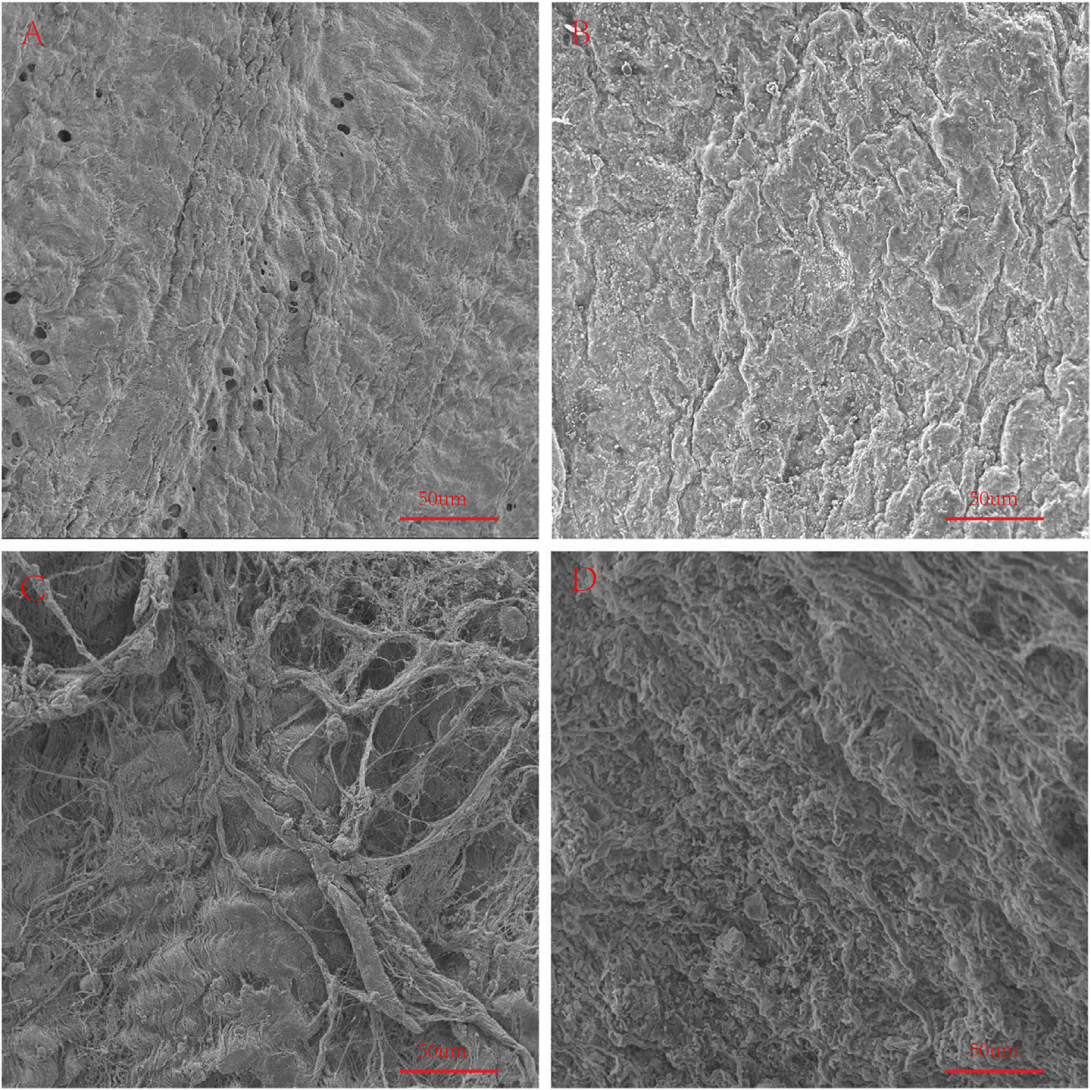
SEM to observe the surface structure of fresh samples. **(A)**, Fresh yak pericardium (YP); **(B)**, Fresh Australian bovine pericardium (AP); **(C)**, Fresh local bovine pericardium (LP); **(D)**, Fresh porcine pericardium (PP). scale bars are 50 um.

**FIGURE 7:**
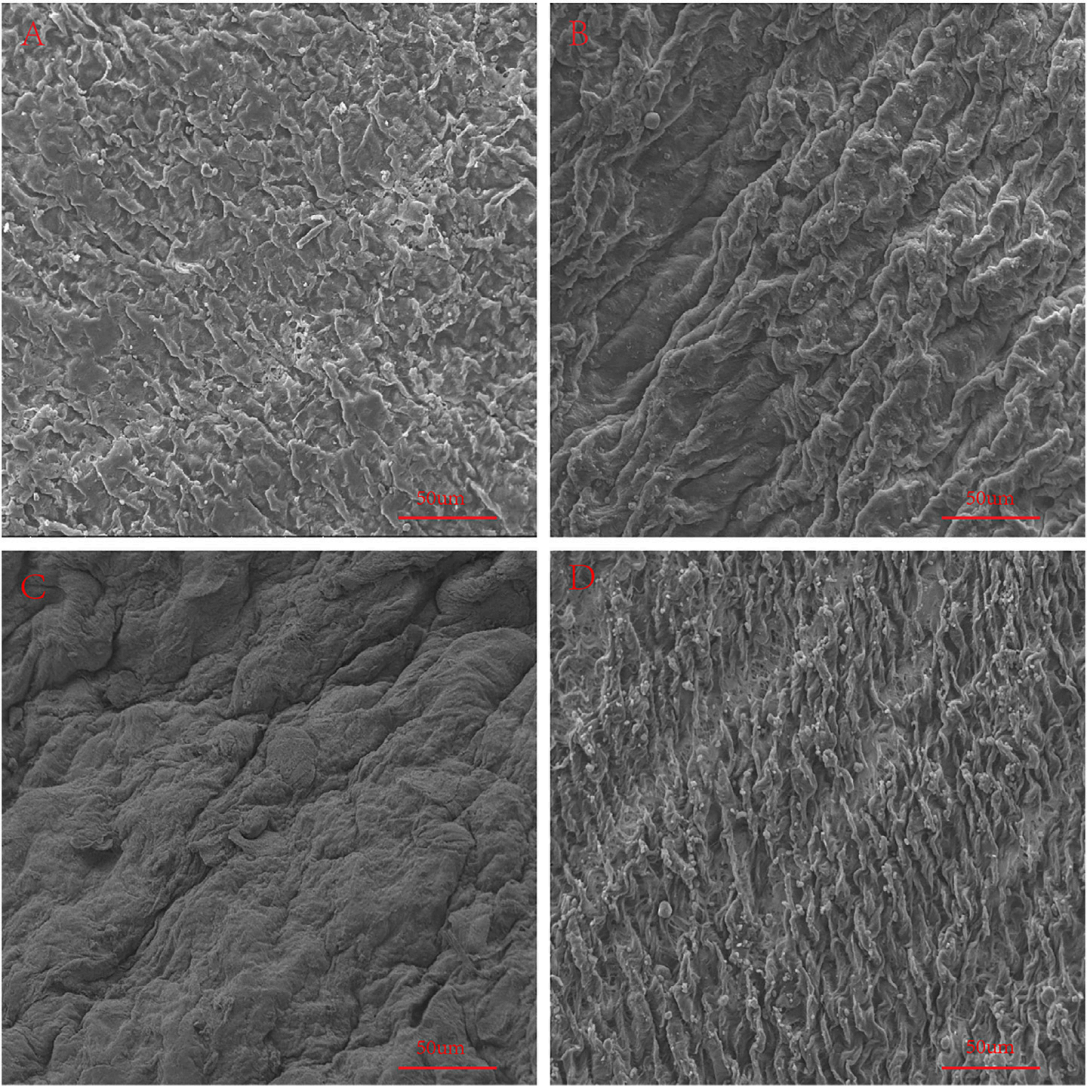
SEM to observe the surface structure of GA-crosslinked samples. **(A)**, GA-crosslinked yak pericardium (GA-YP); **(B)**, GA-crosslinked Australian bovine pericardium (GA-AP); **(C)**, GA-crosslinked local bovine pericardium (GA-LP); **(D)**, GA-crosslinked porcine pericardium (GA-PP). scale bars are 50 um.

We used an electron microscope to observe the surface structure of each pericardium (Figure 6). The fibers of the LP are disordered and sparsely arranged. The fibers of the YP are arranged tightly and more regularly. The fiber structure of fresh PP is arranged irregularly, compared with the fiber arrangement of AP being tighter and more regular. After glutaraldehyde cross-linking, the collagen fiber structure of all pericardia becomes very dense, which is consistent with its mechanical and stained structure (Figure 7).

### Transplantation-Associated Antigen Detection

**FIGURE 8:**
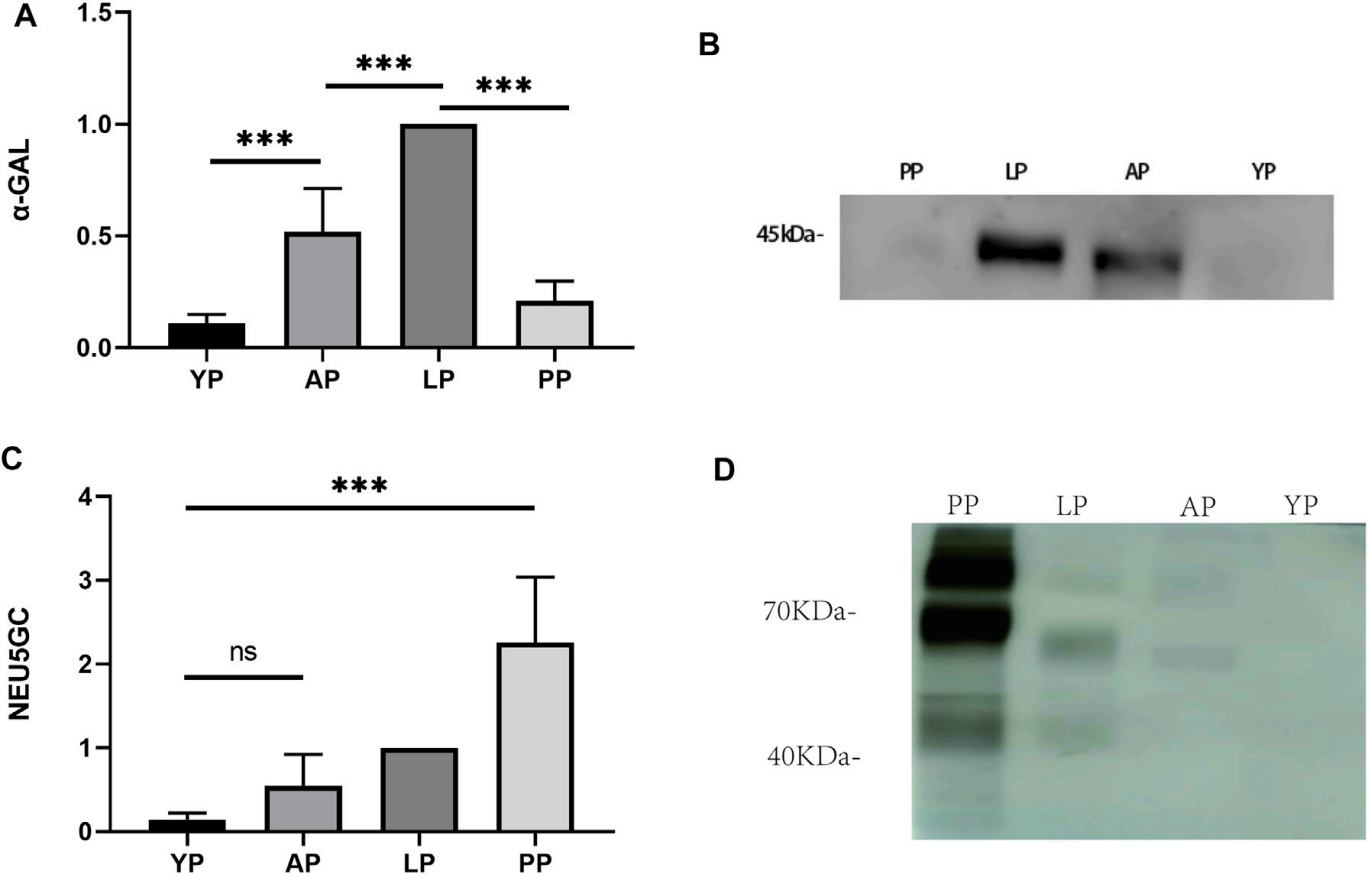
Western-blot to detect transplantation-associated antigens of four different groups of fresh pericardium. Quantitative comparative analysis of α-gal **(A)** and NEU5GC **(C)**; **(B, D)** show that the expression of α-gal and NEU5GC of YP is the least among the four groups of pericardium. (*n* = 3; *p < 0.05; **p < 0.01;***p < 0.001; ns represents no significant difference).

Immunogenicity is one of the primary causes of valve degradation; therefore, we tested the graft-specific antigens of each pericardium. α-Gal is currently one of the most important graft-related antigens (Daly et al., 2009). We used the Western-Blot method to detect the expression of α-Gal in the fresh pericardia of each group. The expression of α-Gal in YP was significantly lower than that of the other groups (Figures 8A–C). We compared the expression levels of NEU5GC in each pericardium. Similar to other literature reports (Byrne et al., 2015), the expression levels of NEU5GC in the PP were significantly higher than that of the other three groups, while the expression levels of NEU5GC in the YP were the lowest (Figures 8B–D). The antigen expression results indicate that YP has lower immunogenicity and is more suitable to produce biological valve materials.

### Folding Experiment

**FIGURE 9:**
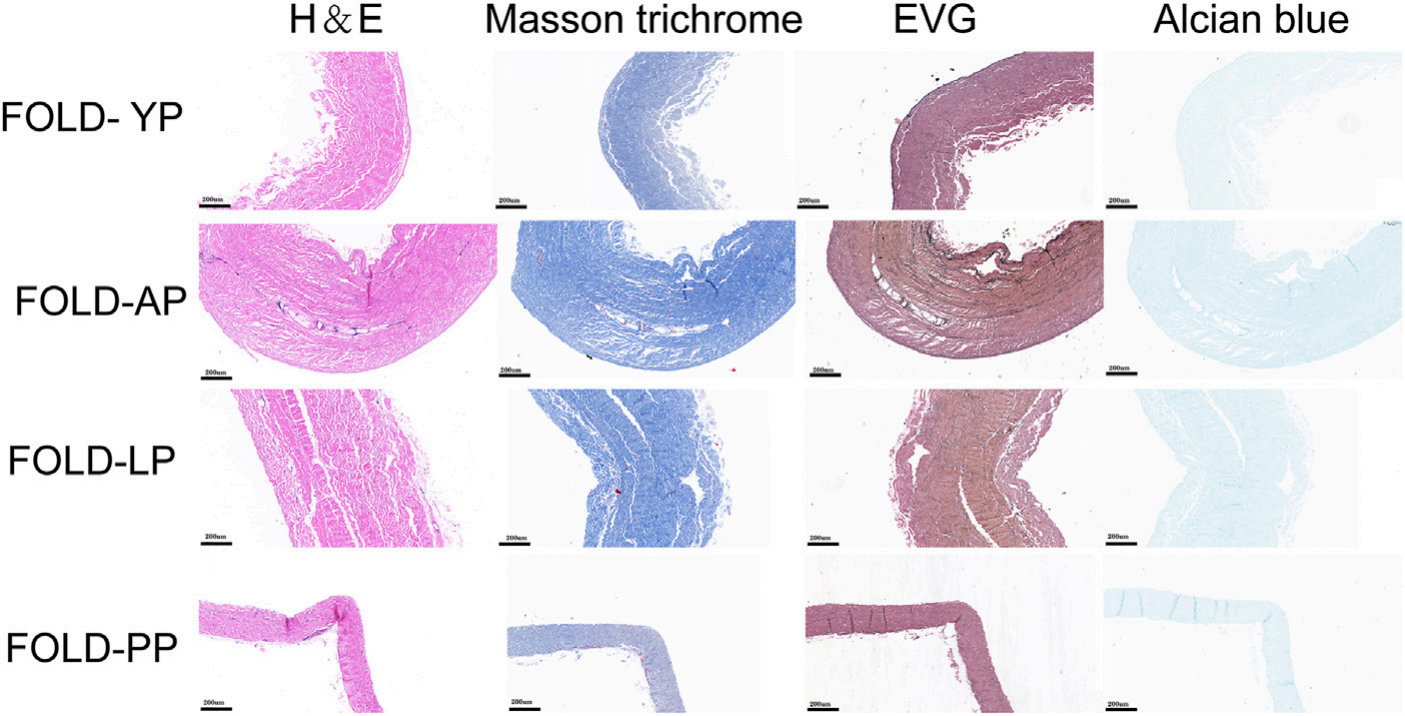
HE staining, MASSON staining, EVG staining, and Alcian blue staining for ECM evaluation of Fold-yak pericardium, Fold-Australian bovine pericardium, Fold-local bovine pericardium, and Fold-porcine pericardium. scale bars are 200 um.

**FIGURE 10:**
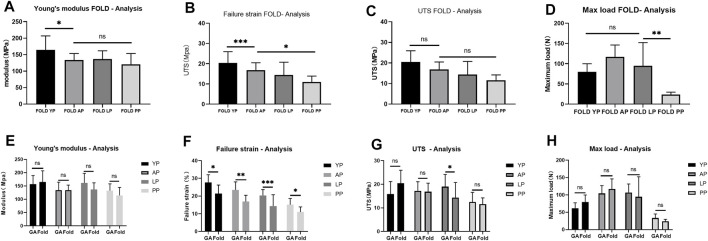
Mechanical performance of Fold-yak pericardium, Fold-Australian bovine pericardium, Fold-local bovine pericardium, and Fold-porcine pericardium And compare the mechanical properties before and after folding. **(A)**, Young’s modulus; **(B)**, Failure strain; **(C)**, The ultimate tensile strength (UTS); **(D)**, Max load. **(E)**, Comparison of Young’s modulus before and after folding. **(F)**, Comparison of Failure strain before and after folding. **(G)**, Comparison of UTS before and after folding. **(H)**, Comparison of Max load before and after folding. we use two-way ANOVA for statistical analysis (*n* = 10; *p < 0.05; **p < 0.01;***p < 0.001; ns represents no significant difference).

**FIGURE 11:**
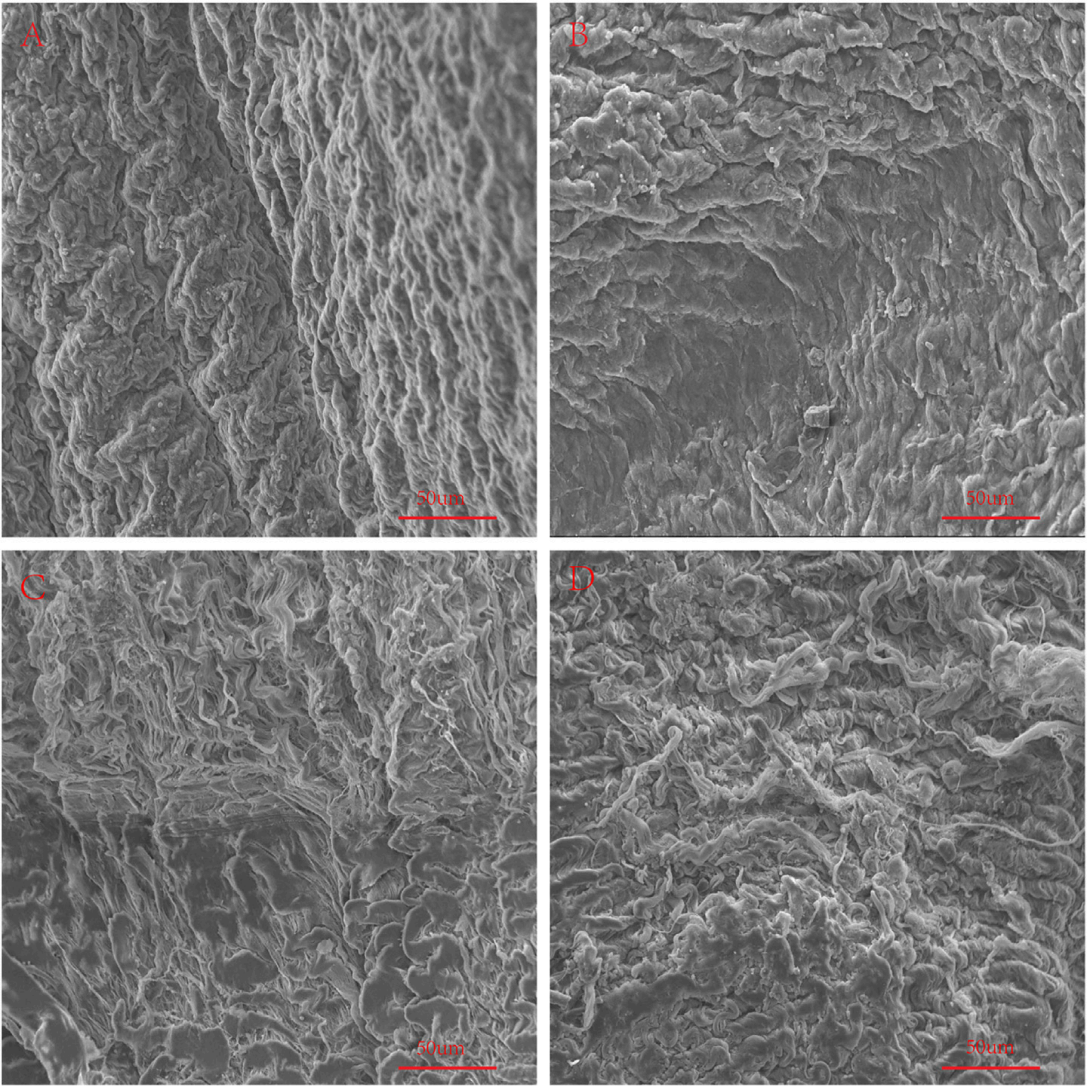
Use SEM to observe the surface structure of Fold samples. **(A)**, Fold-yak pericardium; **(B)**, Fold-Australian bovine pericardium; **(C)**, Fold-local bovine pericardium; **(D)**, Fold-porcine pericardium. scale bars are 50 um.

In order to simulate the material damage that the implantation process of the TAVI may cause, we conducted a folding experiment. We observed the pericardium of the four groups after staining, and it was found that after the folding experiment, the pericardium of the yak showed no noticeable damage. In contrast, the AP and PP showed slight compression damage of the extracellular matrix structure (Figure 9). We performed mechanical testing on the pericardium that had undergone the folding experiment. As presented in Table 1 and shown in (Figure 10), YP has no obvious loss of mechanics compared to the folding experiment. The other groups of pericardia have different degrees of mechanical loss compared to before the folding experiment. Using an electron microscope (Figure 11), YP did not show any apparent fiber breakage, which is consistent with the mechanical results. This shows that YP is a suitable biomaterial for making Transcatheter Heart Valves.

### *In vivo* Biocompatibility and Immune Response

**FIGURE 12:**
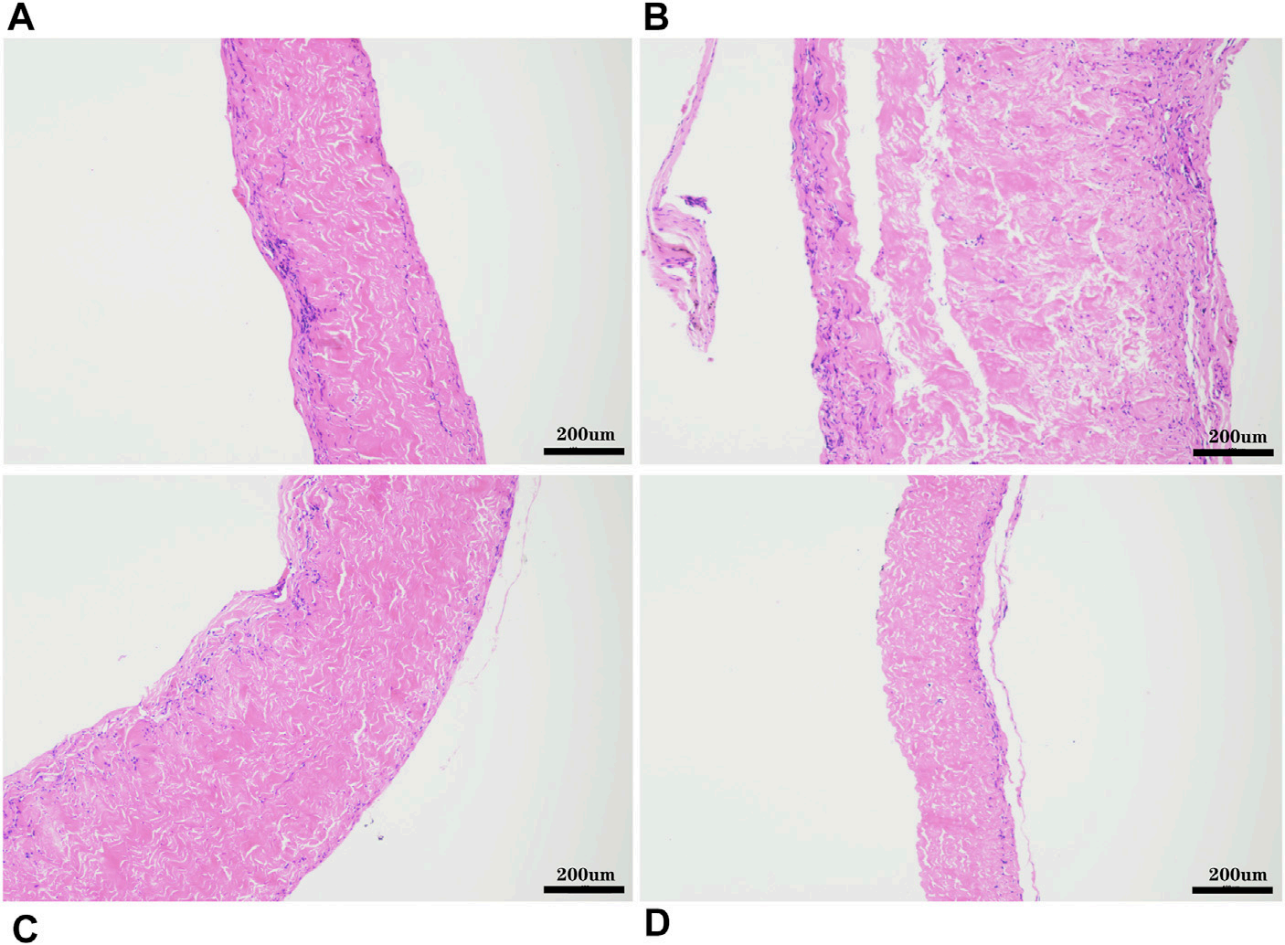
HE staining after 8 weeks of subcutaneous implantation in rats, **(A)**, GA- yak pericardium; **(B)**, GA- Australian bovine pericardium; **(C)**, GA- local bovine pericardium; **(D)**, GA- porcine pericardium. scale bars are 200 um.

The pericardia were harvested after 56 days of subcutaneous implantation and visualized using histological and immunohistochemical staining. The host response to the pericardium’s implants in the H&E stained sections is shown in Figure 12. We found that after the pericardium implantation in each group, the envelopment of a fibrous sac can be observed. We randomly measured the thickness of the fibrous sac and found that the thickness of the fibrous sac of the yak pericardium (22.53 ± 14.18 um) was significantly less than that of the other three groups (44.32 ± 31.78 um; 39.68 ± 21.58 um; 44.66 ± 28.55 um) (Table 1), indicating that the inflammation of the yak pericardium was relatively mild. There were no obvious signs of angiogenesis in the pericardium of the four groups. However, significant fibrous cell deposition in the YP and the AP suggests that extensive recellularization occurs.

**FIGURE 13:**
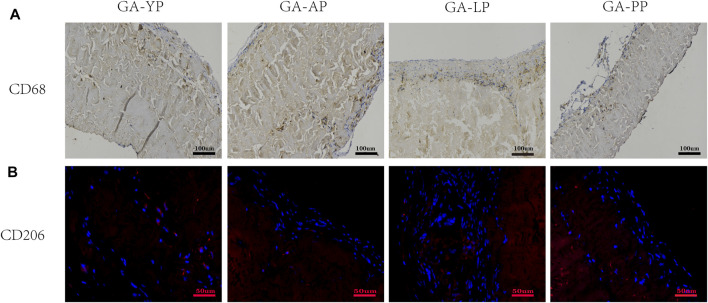
Eight weeks after subcutaneous implantation, immunohistochemistry and immunofluorescence were used to observe the different types of macrophage markers, **(A)** CD68 (M1phenotype brown) scale bars are 100 um. **(B)**, CD206 (M2 phenotype, red) scale bars are 50 um.

We used CD68 immunohistochemically labeled macrophages (M1) to evaluate the host immune response to the pericardial implants. As shown in Figure 13, macrophage infiltration was observed in all pericardial implants; however, CD68^+^ macrophages present in the YP group were less than those in the other three groups. We used CD206 immunofluorescence labeled macrophage’s (M2) to evaluate the anti-inflammatory and regeneration effects of the four groups after pericardial implantation (Figure 13). We found that the expression levels of CD206+ in YP are the highest among the four groups of pericardia.

### *In Vivo* Anti-calcification Assay

**FIGURE 14:**
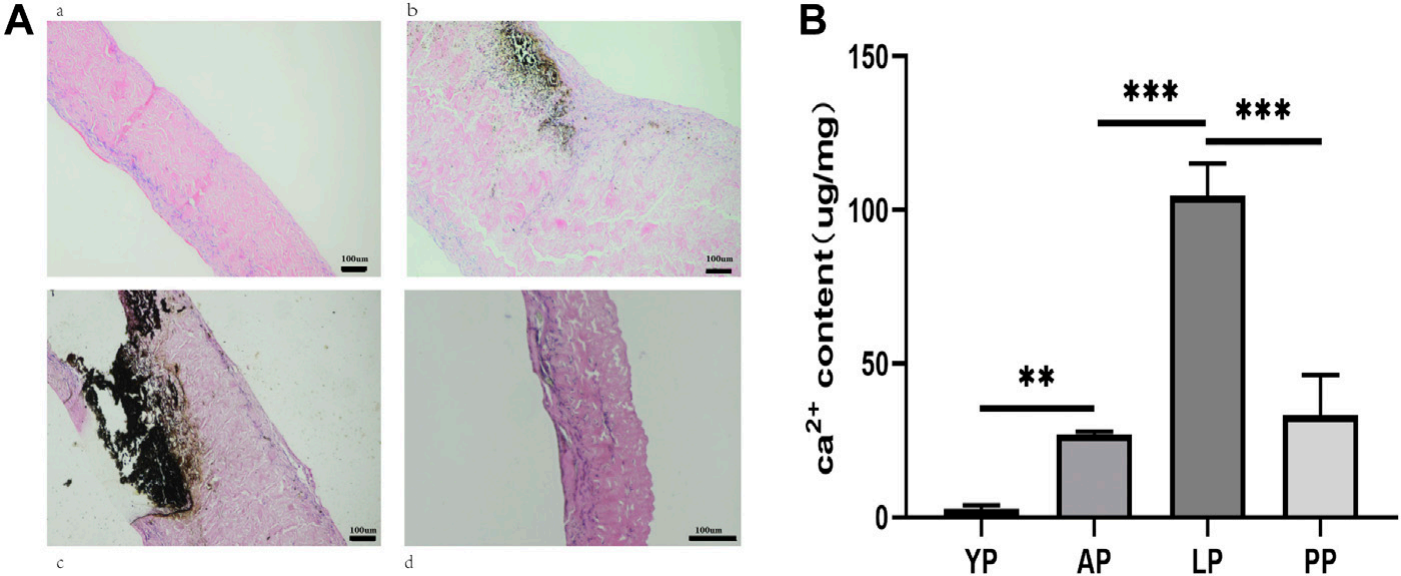
*In vivo* anti-calcification assay of GA-YP, GA-AP, GA-LP and GA-PP after subcutaneous implantation for 8 weeks (*n* = 5 for each group) **(A)**, Von Kossa staining after implantation for 8 weeks. **(B)**, Ca2+ content in the implantation was calculated. scale bars are 100 um.

For the anti-calcification capacity within the subcutaneously implanted pericardia, calcium accumulation spots were stained as black or gray shadows qualitatively using Von Kossa staining (Figure 14A). Higher calcium phosphate levels were detected in the LP and the AP compared to YP, after GA crosslinking for 8 weeks subcutaneous implantation in rats. This tendency agreed with the Ca2+ quantifications (Figure 14B and Table 1). The Ca2+ concentration in the YP group (2.63 ± 1.31 ug/mg) is significantly lower than the LP (104.5 ± 10.7 ug/mg), AP (50.9 ± 13.95 ug/mg), and the PP groups (26.85 ± 10.5 ug/mg) at 8 weeks.

## Discussion

The rate of heart valve disease worldwide has increased year to year with an increase in population aging. Valve replacement surgery accounts for more than 20% of all heart surgery (Iung and Vahanian, 2011). Moreover, people are pursuing an improved quality of life; therefore, biological valves have become more popular as they do not require anti-coagulation therapy and have low bleeding risk. In recent years, the rapid development of TAVI surgery has increased in popularity because it avoids a thoracotomy in order to relieve pain. However, it has significantly increased the demand for the production of artificial heart valves (Hamm et al., 2016). This requires the exploration of novel biological materials that are thinner, more elastic, have superior anti-calcification abilities, as well as possessing a lower immunogenicity. Herein, we introduce a novel biomaterial from natural yak pericardium as a promising alternative for cardiovascular biomaterials, taking advantage of its mechanical properties, low immunogenicity, and excellent calcification resistance. Furthermore, a large number of standardized yak breeding farms and slaughterhouses exist in western China, providing a reliable and sufficient source of yak pericardium.

In this study, we evaluated the macro and micro structural properties of the yak pericardium. We compared the yak pericardia with the current commercially available valve materials. YP’s collagen and elasticity travel more regularly than LP, AP, and PP in terms of the overall structure. YP also has the advantage of thickness compared to LP and AP. It is important to note that the thickness of the pericardium leaflet tissues is of crucial importance for TAVI utility, and the performance and long-term durability of the valve (Cribier et al., 2002). Although PP is slightly thinner than the YP in thickness, the fibers of PP run irregularly, and the material is soft and not plastic enough. The primary components of the extracellular matrix of the pericardia include collagen, elastin, and GAGs (Cramer and Badylak, 2020). Among them, the arrangement and content of collagen and elastin are the important factors affecting the mechanical and thermal stability of the tissue (Cocciolone et al., 2018; Wahart et al., 2019). Due to the regular arrangement of collagen and rich elastin content, YP’s mechanical properties are significantly better than the other groups of pericardia, especially after GA cross-linking; thus increasing its strain failure of YP compared to LP, AP, and PP. In our enzymatic degradation study, YP can prevent collagenase hydrolysis, reflecting its stable structure. The YP has lower water content, meaning that its tissue structure is denser. This may be one of the reasons why YP is thin and maintains its excellent mechanical abilities. We measured the amino acid content of the four groups of pericardia, and found that the content of lysine in YP was significantly higher than in the other pericardial groups**.** The degree of cross-linking also supports the results of the amino acid test. Many studies have found that glutaraldehyde is primarily cross-linked through the lysing amino acid side chain group (Jorge-Herrero et al., 2005). Furthermore, we believe that the cross-linking between YP and glutaraldehyde is more extensive, resulting in improved mechanics and thermal shrinkage. The hydrophobic structure of elastin is the reason for the molecule’s elasticity (Han et al., 2003). YP contains more glycine and valine, suggesting that YP may have better compressive and tensile strength as well as better mechanical properties, which is consistent with our mechanical and folding experimental results. Higher elastin content causes higher fatigue resistance in the yak, meaning better durability and plasticity. In TAVI surgery, a valve consisting of pericardial leaflets mounted on a metal stent must be crimped to a small diameter in order to fit in the deployment of the catheter. After determining the deployment position, a balloon catheter is used to self-inflate to deploy and fix the valve in place. (Berlin et al., 2015) An important limitation of TAVI is the potential damage to the heart valve during the folding and balloon inflation process; therefore, we performed a folding experiment to simulate the folding and inflation process (Munnelly et al., 2012; Jalal et al., 2016). In our folding experiment, the yak pericardium has no apparent structural damage after folding, and there is no mechanical loss. This may be related to the high content of yak elastin, which has better compression resistance and plasticity. These physical factors contribute to the possibility of YP acting as a transcatheter heart valve substitution.

The immunogenicity of the biomaterial is an important factor for the degradation of materials after implantation. α-gal and NEU5GC are recognized as critical transplantation-related antigens (Galili, 2013; Salama et al., 2015; Reuven et al., 2016; Li, 2019), and a large number of studies have confirmed that their expression is significantly related to the rejection and calcification after the implantation of the biomaterial (Reuven et al., 2016), (Manji et al., 2006; McGregor et al., 2011; Byrne and McGregor, 2012; Manji et al., 2012; Byrne et al., 2018). We first verified the expression of α-gal and NEU5GC antigens in fresh tissues of YP, AP, LP, and PP. YP has a lower expression of NEU5GC, and α-gal compared to other pericardia, which is the same result confirmed through subsequent subcutaneous embedding experiments; thus, proving that it is more suitable for valve production. Similar to other research reports, the expression levels of NEU5GC of PP are the highest of all pericardium, meaning that PP will cause a stronger immune response, thereby reducing the durability of the valve material. In the pericardial tissue after subcutaneous implantation in rats, we found that the inflammatory response of the YP is the lowest and is consistent with our *in vitro* antigen test results. A thick fibrous sac means that inflammation is present; therefore, it is difficult to regenerate the fibrous encapsulation. (Thampi et al., 2013) We evaluated the thickness of the fibrous sac of the four groups after pericardial implantation. The thickness of the fibrous sac of YP was thinner than that of the rest of the samples; therefore, YP has a lower inflammatory response as well as the possibility of recellularization. We observed that all four sample groups lacked angiogenesis after implantation.Angiogenesis are related to inflammation (Thampi et al., 2013). Since our four groups of samples are cross-linked by glutaraldehyde, and glutaraldehyde cross-linking can reduce inflammation reaction (Manji et al., 2006).we did not find the angiogenesis, which may be related to the inhibitory inflammation effect of glutaraldehyde cross-linking,which may have a positive effect on regeneration. Macrophages are associated with inflammation and regeneration, in which M1 macrophages are closely related to inflammatory response, while M2 macrophages are closely related to anti-inflammatory and regeneration responses (Klopfleisch, 2016). In the subcutaneous implantation experiment, YP has the lowest M1 positive and the highest M2 positive expression, indicating that YP has a lower inflammatory response. Tyrosine plays an important role in immunogenicity, and some studies have reported a correlation between tyrosine and biological valve calcification (Lee et al., 2017). The tyrosine content of the yak is significantly lower than that of the bovine and porcine pericardium. Therefore, we can infer that the low immunogenicity of the yak pericardium may be due to its amino acid composition. This needs to be further investigated.

Calcification is currently one of the most severe problems leading to the failure of biological valve materials. We performed a subcutaneous embedding model to compare the calcification levels of each pericardium. Interestingly, the calcification level of the yak pericardium after 56 days of subcutaneous implantation showed significantly lower levels of calcification compared to the other groups. This may be due to the high degree of cross-linking of GA and the related low immunogenicity. This specific mechanism needs to be further studied.

In addition, yak-derived materials have two other advantages. First, it can avoid some zoonotic diseases such as prion disease. Second, it is more acceptable to people with religious beliefs against the use of porcine materials.

YP also has its limitations. For example, it is a very regional species, and it is challenging to promote worldwide. Moreover, the YP’s low immunogenicity requires further research to verify its results and mechanism. The mechanism of YPs anti-calcification ability is still unclear, and further research is needed to verify. In addition, we have not conducted large animal experiments on blood contact, which requires further research. Our current study is still limited to basic biomaterial sheets and has not been sewn into a valve. Dynamic tests including fatigue tests are an indispensable evaluation of surgical biological or transcatheter heart valves and are a necessary means to confirm the potential of yak pericardium-derived materials used as heart valve leaflets and are the focus of our future research.

## Conclusion

We compared a new type of natural material, yak pericardium, which has excellent natural tissue structure, mechanical properties, and has suitable thickness for making a transcatheter heart valve. Through folding simulation experiments, it was found to have excellent compressive properties. We innovatively verified that the YP has low innate immunogenicity, and excellent anti-inflammatory and anti-calcification effects determined via a subcutaneous embossing model in rats. Therefore, we demonstrated that YP materials have significant potential as a cardiovascular biomaterial in the production of transcatheter heart valves.

## Data Availability

The raw data supporting the conclusion of this article will be made available by the authors, without undue reservation.
