# Supplementary material for: Yak Pericardium as an Alternative Biomaterial for Transcatheter Heart Valves
Source: Front Bioeng Biotechnol. 2021 Nov 8;9:766991. doi: 10.3389/fbioe.2021.766991 (PMC8607193; doi:10.3389/fbioe.2021.766991)
Supplement: Supplementary file 1 [file Table1.docx]

|  | YP | AP | LP | PP |
| --- | --- | --- | --- | --- |
| L−Tryptophan(%) | 0.3 | 0.2 | 0.8 | 0.4 |
| L−Phenylalanine(%) | 8.3 | 3 | 5.2 | 3.8 |
| L−Valine(%) | 11.1 | 5.3 | 8.5 | 6.5 |
| L−Methionine（%） | 0.8 | 1.7 | 2.6 | 2.4 |
| 4−Aminobutyric acid（%） | 0.3 | 0.5 | 0.5 | 0.3 |
| L−Tyrosine（%） | 1.2 | 1.8 | 4.5 | 3 |
| L−Proline（%） | 1.4 | 0.8 | 1.5 | 2.5 |
| L−Alanine（%） | 24.5 | 7.5 | 12.8 | 13.4 |
| Glycine（%） | 13.5 | 20.3 | 11.3 | 9.9 |
| L−Glutamic acid(%) | 10 | 31.8 | 21 | 21 |
| L−Threonine(%) | 1.2 | 2 | 2 | 3 |
| 4−Hydroxyproline(%) | 0.6 | 2 | 0.6 | 1 |
| L−Aspartic acid(%) | 0.8 | 1.1 | 2.4 | 2.9 |
| L−Glutamine(%) | 1.7 | 2.4 | 2 | 2.2 |
| L−Serine(%) | 1.4 | 1.5 | 2.7 | 4 |
| L−Citrulline(%) | 1.3 | 2.7 | 3.8 | 2.8 |
| L−Arginine(%) | 2.6 | 4.2 | 3.4 | 6.1 |
| L−Lysine(%) | 17.5 | 7.1 | 10.8 | 11.2 |
| L−Histidine(%) | 0.3 | 0.5 | 1.1 | 1.4 |
| L−Ornithine(%) | 0.9 | 2.3 | 1.9 | 1.9 |
| 5−Hydroxylysine(%) | 0.3 | 1.2 | 0.6 | 0.4 |

Table.S1. Amino acid content detection
