# Supplementary figures and images for: Yak Pericardium as an Alternative Biomaterial for Transcatheter Heart Valves
Source: Front Bioeng Biotechnol. 2021 Nov 8;9:766991. doi: 10.3389/fbioe.2021.766991 (PMC8607193; doi:10.3389/fbioe.2021.766991)

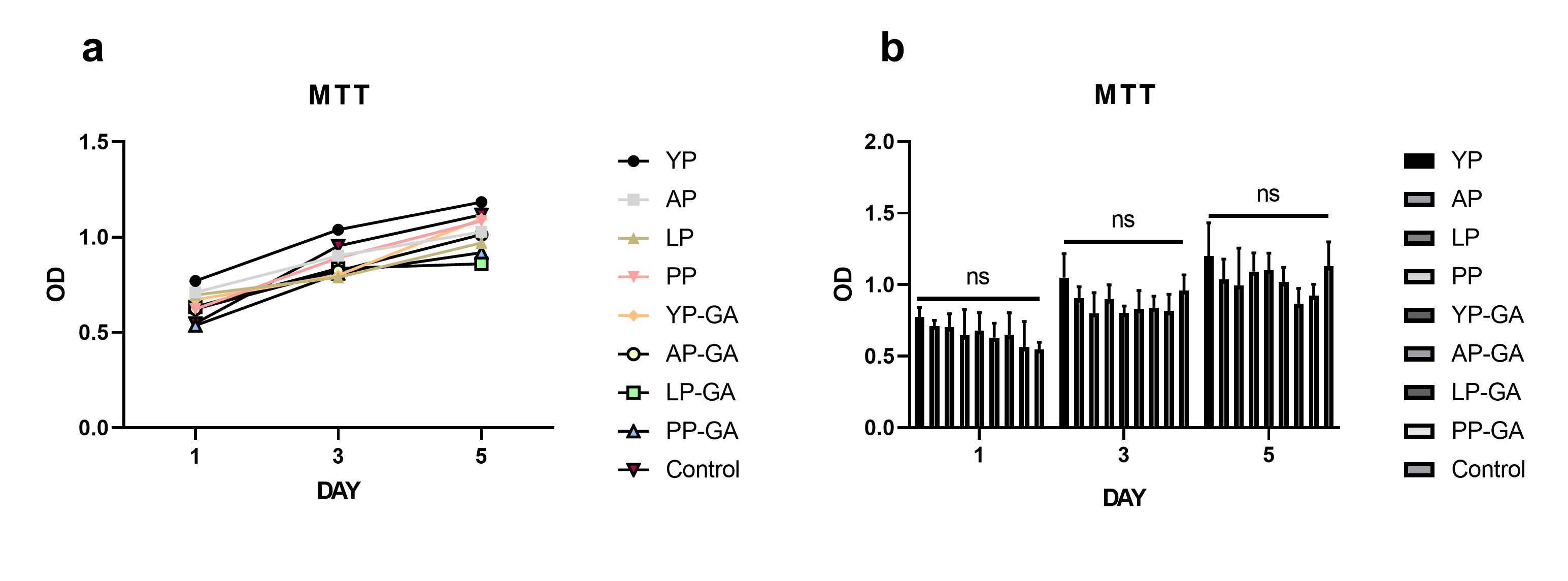

Supplement: Supplementary file 2 [file Image1.TIF]
